# DNA Mismatch Repair Deficiency Detection in Colorectal Cancer by a New Microsatellite Instability Analysis System

**DOI:** 10.1007/s12539-020-00358-8

**Published:** 2020-01-25

**Authors:** Shafei Wu, Xiaoding Liu, Jing Wang, Weixun Zhou, Mei Guan, Yuanyuan Liu, Junyi Pang, Tao Lu, Liangrui Zhou, Xiaohua Shi, Huanwen Wu, Zhiyong Liang, Xuan Zeng

**Affiliations:** 1 Department of Pathology, Molecular Pathology Research Center, Peking Union Medical College Hospital, Chinese Academy of Medical Sciences (CAMS), Beijing, 100730 China; 2 Department of Medical Oncology, Peking Union Medical College Hospital, Chinese Academy of Medical Sciences, Beijing, China

**Keywords:** Mismatch repair deficiency, Microsatellite instability, Colorectal cancer

## Abstract

**Background:**

Although microsatellite instability (MSI) is most commonly detected in colorectal cancer (CRC), improvement in MSI analysis method can always help us better assessing MSI phenotypes and gaining useful information in challenging cases. The purpose of current study is to explore whether the ProDx® MSI analysis System (ProDx® MSI) can improve MSI classification in CRC.

**Methods:**

We compared the MSI profiles of 97 FFPE samples from CRC patients by ProDx® MSI with Promega MSI analysis System 1.2 and NCI panel. The result is then confirmed by IHC test, which evaluate MMR protein expression. Furthermore, next generation sequencing was performed to double confirm the specimens with discordant results.

**Results:**

Among the total 97 CRC cases, 35 were scored as MSI-High by ProDx® MSI, Promega MSI analysis System 1.2, and NCI panel simultaneously. Three extra MSI-High cases were identified by ProDx® MSI. These three cases were classified as MSI-Low by NCI panel, while two of these as MSI-Low, and 1 as MSS by Promega MSI analysis System 1.2. ProDx® MSI had higher concordance with IHC detection compared with Promega MSI Analysis System 1.2 and NCI panel at 99.0%, 96.9%, and 95.9%, respectively. The ProDx® MSI distinguished MSI status with 100% sensitivity and 98.4% specificity. Our data showed that MSI-High phenotype occurred most frequently in tumor development stage I and stage II.

**Conclusions:**

The colorectal cancer can be classified according to MSI status accurately by ProDx® MSI. More cases with MSI-High feature may be revealed by ProDx® MSI than by previous test systems in colorectal cancer.

## Introduction

Microsatellite instability (MSI), identified by changes in the length of short tandem repeats of microsatellite markers in tumor DNA, is caused by an impaired DNA mismatch repair (MMR) system that fails to repair DNA replication error during tumor development [1]. MSI occurs relatively frequently and accounts for 10–15% of colorectal, stomach, and endometrial cancers, while it is less frequent in other solid tumors [2]. The MSI status has broad clinical implications: (1) It is considered a hallmark for Lynch syndrome. (2) Patients with MSI tumors are known to have better prognosis of disease than those with microsatellite stable (MSS) lesions [3]. (3) Patients with MSI tumors are less responsive to 5-FU adjuvant chemotherapy [4]. (4) MSI status may act as a biomarker for immunotherapy treatment [5].

Historically, two distinct strategies have been used to determine MSI status and MMR function: (a) MSI analysis to determine the instability in microsatellite markers and (b) immunohistochemistry (IHC) to determine the loss of one or more MMR protein expression. In 2008, Shia and Zhang reviewed the pros and cons of each strategy [6, 7]. Both methods, especially MSI analysis, have undergone significant changes since then. Early in 1997, a National Cancer Institute (NCI) workshop proposed the *Bethesda Guidelines* recommending a panel of five microsatellite markers (NCI panel with 2 mononucleotide repeats and 2 dinucleotide repeats) for MSI detection and tumor classification in colon cancer [8]. In 2004, NCI published Revised *Bethesda Guidelines* recommending an additional marker panel of all mononucleotide satellite markers to further increase sensitivity [9]. A commercial MSI analysis system from Promega Corp containing five mononucleotide repeats demonstrated improved sensitivity, specificity, and popularity [10, 11]. Furthermore, studies showed that tumors with MSH6 deficiency, or certain tumor types such as endometrial cancer, were difficult to assess by the dinucleotide repeat markers [12, 13]. The existing MSI analysis markers were also found less sensitive in early onset cancer [14]. MSI is a progressive phenomenon that MSI phenotype might change during the cancer development.

To further improve the assay sensitivity, Bacher et al. screened a class of very long mononucleotide repeat markers of 40–60 bp, which are distinctly longer than the traditional mononucleotide repeats. The frequency of mutation in mononucleotide repeats increases exponentially with accumulating number of repeating units, which leads to increased sensitivity of MSI detection. Their study showed that employing the long mononucleotide repeat markers improved detection sensitivity and specificity compared with the commercially available five mononucleotide repeat panel and NCI panel in early colorectal lesions and other tumors [15].

In this report, we compared the new ProDx® MSI Analysis System (ProDx® MSI), containing the long mononucleotide repeats (LMR), against the commercially available MSI analysis system version 1.2 (MSI 1.2), the NCI panel, and the MMR—IHC detection methods. Our findings suggested that the ProDx® MSI increased the detection sensitivity of MSI-High in colorectal cancer samples with easier phenotype determination. This enhanced detection sensitivity for the ProDx® MSI may help labs identifying true MSI-High phenotypes in many cancer types to guide proper clinical treatment.

## Method

### Tissue Specimens

Total 97 cases of formalin-fixed paraffin-embedded (FFPE) specimens from colorectal cancer with a complete medical history archived in Peking Union Medical College Hospital were analyzed retrospectively.

### IHC Analysis

The IHC study on MMR protein (MLH1, PMS2, MSH2, and MSH6) expression in tumor tissue was carried out on 4-µm-thick FFPE sections using manufacturer-recommended automated staining protocols on a BOND-III Fully Automated IHC and ISH Stainer (Leica Microsystems; Melbourne, Australia). The MMR antibodies (MLH1, PMS2, MSH2, and MSH6) used in this study are clones ES05, MOR4G, 25D12, and PU29, respectively (Novocastra; New Castle, UK).

### Microsatellite Analysis

DNA was extracted from macro-dissected FFPE tumor tissue slides and from matching normal FFPE tissue by Maxwell 16 FFPE Tissue DNA Purification Kit (Promega, Madison, WI). The DNA concentration was then quantified using a Nanodrop (Thermo Scientific, Wilmington, DE). Approximately 5–10 ng of purified DNA was used for MSI analysis with three different microsatellite testing panels: (1) ProDx® MSI containing eight mononucleotide repeat markers with four new long mononucleotide repeats (BAT-52, BAT-56, BAT-59 and BAT-60) and four traditional markers (NR-21, BAT-25, BAT-26, and MONO-27) and two additional pentanucleotide repeats Penta C and Penta D for sample identification (Shanghai Promega), (2) MSI 1.2 (Shanghai Promega) containing five traditional mononucleitide repeats BAT-25, BAT-26, NR-21, NR-24, and MONO-27 and two pentanucleotide repeats Penta C and Penta D for specimen identification (Promega, Madison), (3) the NCI panel (also known as the Bethesda panel) consisting of two mononucleotide repeats BAT-25 and BAT-26 and three dinucleotide repeats D2S123, D5S346, and D7S250 [15]. PCR products were separated on a 3500Dx Genetic Analyzer with POP7 polymer and 50-cm capillary configuration. The data were analyzed with GeneMapper 5.0 Software (Applied Biosystems). MSI determination: allelic sizes for matching tumor and normal specimens were compared, and the marker was defined as MSI unstable if there was a shift of three base pairs in the cancer allele. Specimens were categorized into MSI-High (MSI-H) when two or more microsatellite markers were unstable, MSI-Low (MSI-L) when one marker was unstable, or MSI stable (MSS) when there was no any unstable marker.

### Next Generation Sequencing Analysis

MMR gene mutations were further analyzed by NGS for case 50N/T with MSI-H feature identified by 3 methods of MSI assays but with intact MMR protein IHC staining, as well as cases of 46N/T, 67N/T, 138N/T, 165N/T, 75N/T, 132N/T and 55N/T which with MSI-L feature tested by ProDx® MSI but with MSS phenotype detected by MSI NCI panel and MSI 1.2. Purified FFPE DNA extracted from macro-dissected FFPE tumor tissue slides and matching distal peritumoral tissue was quantified and analyzed by next generation sequencing (NGS). The sequencing panel included MLH1, MSH2, MSH6, PMS2, and 19 other genes that are indicators for familial inherited risk of solid tumor.

### Data Analysis

The sensitivity and specificity for the recognition of MSI-H phenotype were measured using IHC test, which is the gold standard, for MLH1, PMS2, MSH2, and MSH6 protein expression in tumors. Sensitivity and specificity for discriminating MSI-H feature in the samples were assessed using the following formulas:

- Sensitivity = true positives/ (true positives + false negatives).
- Specificity = true negatives/(true negatives + false positives).
- Concordance = (true positives + true negatives)/total samples.

True positives were demonstrated MSI-H with loss of MMR expression by IHC (or germline MMR gene mutation). True negatives were MSI stable with normal MMR expression by IHC. False positives were MSI-H with intact MMR protein expression by IHC. False negatives were MSI stable with lack of MMR founction by IHC (or germline MMR gene mutation).

## Results

### Study Patients and Specimens

Ninty-seven patients with colorectal cancer were included in the study in Table 1.

### MSI Analysis in Colorectal Cancer

We conducted MSI analysis on 97colorectal cancers with the ProDx® MSI, the MSI 1.2, and the NCI panel. The results were cross referenced with IHC for MLH1, MSH2, MSH6, and PMS2 protein expressions in Table 2. Of 97 colorectal cancers, 37 samples were scored as MSI-H via the ProDx®MSI, compared to 35 by MSI 1.2 and 34 by the NCI panel. In all cohorts, three MMR protein deficient (dMMR) cases, 43N/T, 36N/T, and 163N/T, were detected as MSI-L by the NCI panel. Two of these, 36N/T and 163N/T, were tested as MSI-L by MSI 1.2. All three were correctly distinguished as MSI-H with the ProDx® MSI. The data indicated that ProDx® MSI detected more MSI-H phenotypes in colorectal cancers compared with the two historic MSI analysis systems. The extra MSI-H detection was a result of instability in the long mononucleotide repeat markers BAT-52, BAT-56, BAT-59, and BAT-60.

When MSI-H samples were grouped by the disease stages, our data indicated that MSI-H phenotype occurred most frequently in tumors at development stage I and stage II. When grouped by the development stage, earlier stage cancer group showed higher percentage of MSI-H phenotype compared with the later stage groups. MSI-L phenotypes also occurred in higher percentage in stage I and II cancers in Table 3. In addition, germline sequencing was conducted on nine cases who were confirmed as Lynch syndrome positive [16]. All Lynch syndrome cases exhibited MMR protein expression loss by IHC and MSI-H by ProDx® MSI test.

MSI results were detected by ProDx® MSI. Percentage was calculated within each disease stage.

### IHC Analysis and NGS Analysis

IHC analysis for MLH1, PMS2, MSH2, and MSH6 protein expression was performed on all samples. Compared with MMR-IHC detection, the ProDx® MSI showed 100% sensitivity and 98.4% specificity, where it detected MSI-H on all dMMR case and two more MSI-H on pMMR cases. The MSI 1.2 showed 94.4% sensitivity and 98.4% specificity; the NCI panel showed 91.7% sensitivity and 98.4% specificity in colorectal cancers.

**Fig. 1:**
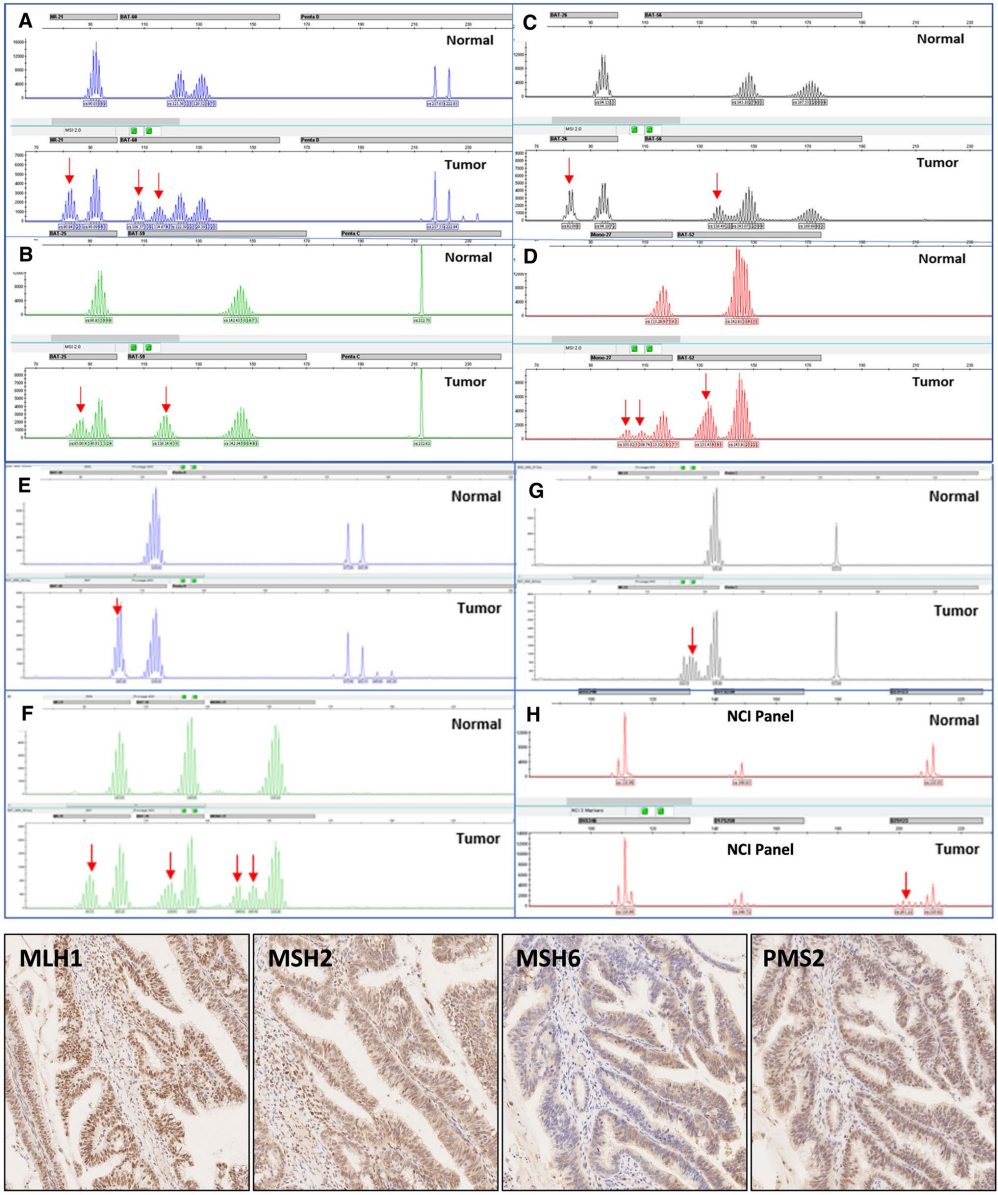
MSI and IHC results for case 50N/T. Panels A-D were MSI test electropherograms using ProDx® MSI kit. Panels E-F were MSI test electropherograms using MSI1.2 kit. Panel H was MSI test electropherogram with three dinucleotide repeat markers from NCI panel. For each panel, top trace was from normal tissue, and bottom trace was from the tumor tissue. Red arrows indicated unstable markers. Bottom panels were immunohistochemistry staining for 4 MMR proteins in tumor tissue with anti-MLH1, MSH2, MSH6, and PMS2 antibodies, respectively

Our data indicated one discordance between ProDx® MSI and MMR-IHC detection. Case 50N/T with wild type MMR protein expression by IHC analysis showed MSI-H in all three MSI analyses in Fig. 1.

To verify the discordant results between ProDx® MSI and MMR-IHC detection, case 50N/T was sequenced for MLH1, PMS2, MSH2, and MSH6 gene exon mutations in matching normal and tumor tissues . Case 50N/T had possible pathogenic mutation (c.3438+1G>A) in MSH6 gene with increased disease risk in both normal tissue and tumor tissue.

Pathological MMR gene mutations were not found in cases of 46N/T, 67N/T, 138N/T, 165N/T, 75N/T, 132N/T and 55N/T by NGS assay.

### Characterization of ProDx^®^ MSI in the Chinese Population

**Fig. 2:**
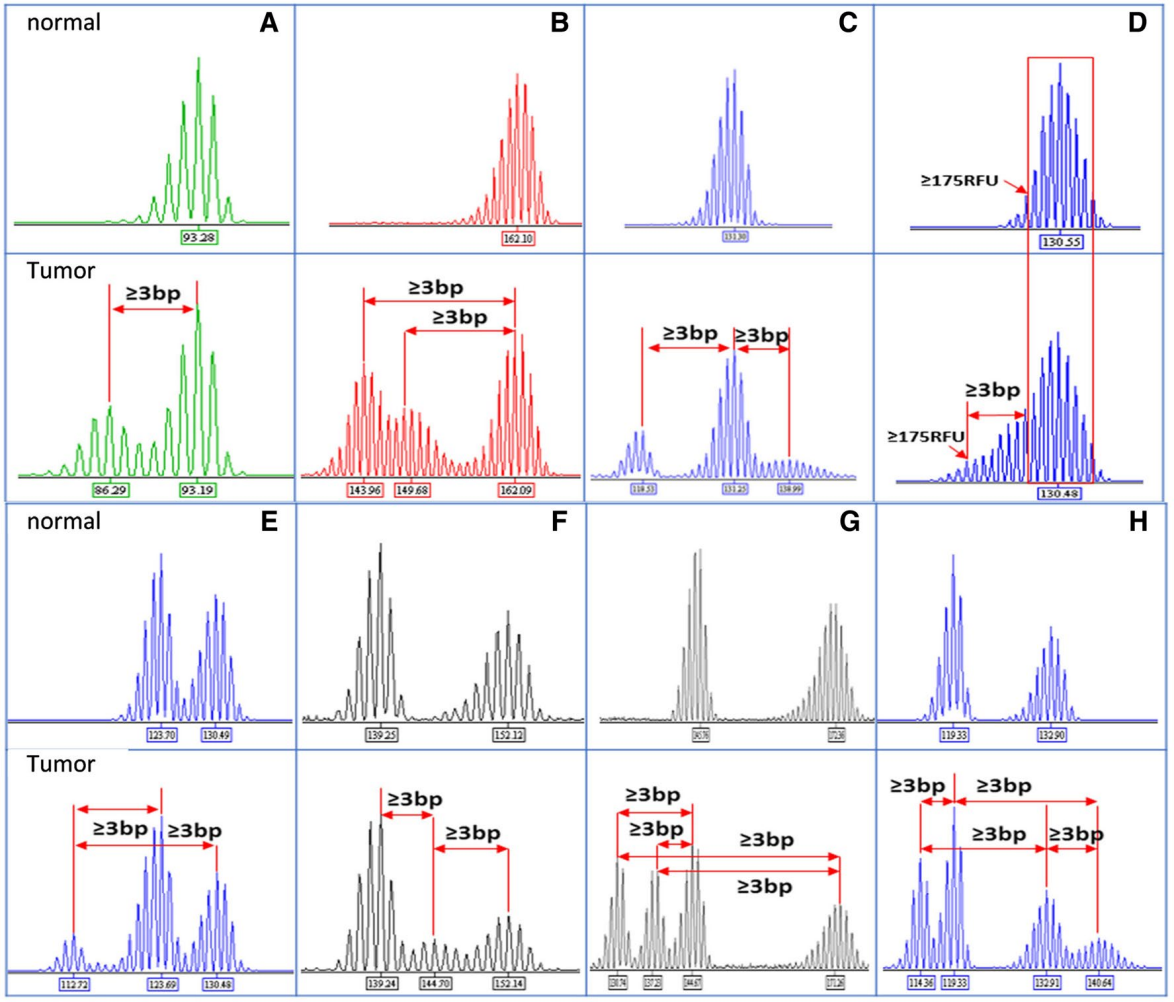
Example of unstable marker profile in MSI-H samples. Top panels displayed the marker profiles for the normal tissue, and bottom panels were the marker profiles for the matching tumor samples

Microsatellite marker instability is commonly defined as the presence of new ≥ 3 bp alleles in the tumor specimen compared to that in matching normal sample. In addition to the presence of new “hand shape” peaks, our study observed markers with ≥ 3 bp extended “shoulder” and defined these as marker unstable in this study in Fig. 2 (Panel D). The definition was consistent with the “subtle” marker changes shown in 2014 College of American Pathologists (CAP) survey summary [11].

In addition to different MSI analysis systems, we also evaluated the individual marker’s sensitivity and specificity in comparison with MMR-IHC phenotype in Table 4. The LMR markers (BAT-52, BAT-56, BAT-59, and BAT-60) and the traditional mononucleotide repeat markers (NR-21, NR-24, BAT-25, BAT-26, and MONO-27) had similar detection sensitivity and specificity in CRC compared with IHC data. However, the mononucleotide repeat markers were significantly more sensitive than the dinucleotide repeat markers (D2S123, D5S346, and D7S250) in this study.

**Fig. 3:**
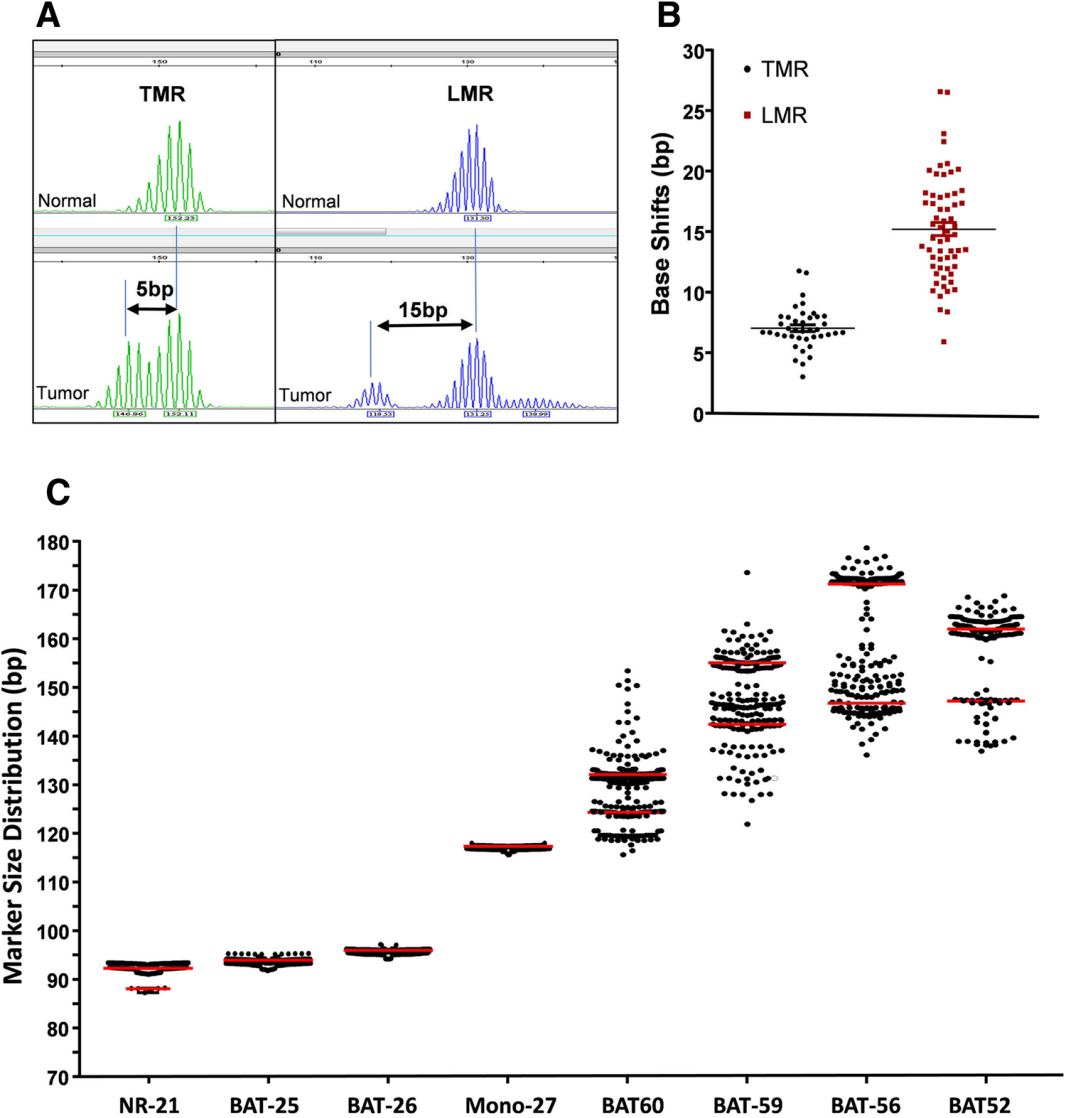
Base changes in long vs traditional MSI markers in MSI-H cases and size distribution of the ProDx® MSI analysis marker in 160 cases of normal tissues in Chinese. **a** Electropherogram example of shifted marker for the long mononucleotide repeat (LMR) marker and the traditional mononucleotide repeat (TMR) marker of the same paired sample. **b** Scatter graph of shifted bases for each unstable marker. **c** Each dot represented the highest allele location for the marker from one proband. Red line represented the median size for the tallest allele in the marker cluster

The long mononucleotide repeat markers (BAT-52, BAT-56, BAT-59, and BAT-60) were compared with the traditional mononucleotide repeat markers (NR-21, NR-24, BAT-25, BAT-26, MONO-27). When marker was unstable, the allele size change for the long markers were greater than the short markers in MSI 1.2, averaging 15 bp vs 7 bp, respectively ( Fig. 3a/b), *p* < 0.0001, student *t *test). The larger changes made marker scoring easier.

The long MSI marker allele frequency in the Chinese population is unknown. We summarized the allele frequency from 160 cases of normal tissues from different Chinese individuals. Most of the short markers NR-21, BAT-25, BAT-26, and MONO-27 were homozygous (Fig. 3c) . NR-21 showed 3.7% heterozygosity in the Chinese population. In addition, the size distribution for the short marker sizes were very stable in the population. The median size for NR-21 was 92 bases, with the heterozygosity peak at 88 bases in the ProDx® MSI and under the tested condition. The median sizes for BAT-25, BAT-26, and MONO-27 were 94, 95, and 117, respectively, under the current condition. In contrast, the long markers BAT-52, BAT-56, BAT-59, and BAT-60 showed a high degree of heterozygosity at 21%, 42%, 63%, and 64% of the population, respectively.

## Discussion

MSI analysis method and IHC method for four MMR protein were reported as equally effective in detecting DNA mismatch deficiency in CRC [6, 7]. Our data supported that the ProDx^®^ MSI had very high concordance with MMR-IHC analysis. In this study, we identified 1 discordant cases between MSI analysis and MMR in 97 samples. The *MLH1, MSH2*, *MSH6*, and *PMS2* gene exon mutations in the matching normal and tumor tissues were sequenced by next generation sequencing. The sequence results for case 50N/T indicated possible pathogenic mutations in the *MSH6* gene in both tumor and normal tissue, which supported the MSI-H results shown from MSI testing. However, the mutation was classified as possibly pathogenic with increased risk level. The sequencing data could not conclude that IHC results were false. The sequencing results indicated the potential limitations of NGS in determining MMR mutation’s pathological function. Cases of 36N/T and 163N/T showed MSI-L feature detected by MSI 1.2 and NCI panel detection. But their MSI-H feature was revealed by the new panel of microsatellite markers, which confirmed by IHC results with defective protein expression. It suggested that ProDx^®^ MSI had higher sensitivity than the traditional loci. In our study, both MSI analysis method and IHC method could effectively identify MSI-H or mismatch deficient cases in CRC. Although the two methods used different technology platforms, each has advantages and disadvantages in clinical labs. In general, IHC method can identify the missing protein caused by mutations that lead to truncation, protein instability, or promoter silencing. The method may not be effective on missense mutation with intact antigen. Reports showed cases of false-normal staining for MLH1 with truncating and large in-frame deletions in *MLH1* gene [6]. PCR-based MSI analysis is a functional test with reported reproducibility close to 100%. It can detect dMMR tumor with genetic defects beyond four MMR genes. However, tumor-specific MSI sensitivity is still unknown [7].

As in the previous report of improved detection sensitivity by the long mononucleotide repeat markers in colon polyps, our study on colorectal cancer patients indicated that the ProDx^®^ MSI has minor improvement in detection sensitivity. This might be due to different tumor types or stage effect. In fact, our ongoing study indicates that the ProDx^®^ MSI can detect significantly more MSI-H phenotypes in endometrial cancer and several other cancer types (data not shown). This system may have strong advantage in other tumors.

More interestingly, ProDx^®^ MSI detected not only more MSI-H samples in CRC but also more MSI-L types with a LMR marker instability, such as BAT-52, BAT-56, and BAT-59 (Table 2). The MSI-L phenotype was very reproducible. In the past, the MSI-L tumor was often considered as MSS because those tumors were not linked to Lynch syndrome [15, 17]. Our data showed three MSI-L cases detected by NCI panel were MSI-H by the ProDx^®^ MSI and the MSI 1.2. Those three cases were also identified as mismatch repair deficient by IHC. Our data indicate that MSI-L cases by the NCI panel could in fact be MSI-H with a more sensitive detection system, which implied a biological relevance for MSI-L in tumor. Other studies suggested that MSI-L phenotype might reflect the early phase of losing mismatch repair function during tumor development [18, 19]. Our data, although limited by sample size, showed the MSI-L occurred more often in stage I and II of colorectal cancer (Table 3), which implied its relevance in the early stage of cancer development. In addition, MSI-L phenotype was also observed in many other cancer types. Further research is required to explore the true biological significance for a MSI-L type tumor.

In addition to MSI-L, our data also showed that MSI-H phenotype occurred more frequently in the early stages (I/II) of cancer development (Table 3). We did not observe MSI-H in stage IV colorectal cancer. Although, there was a lower presentation of stage IV samples, the observation was consistent with other reports [20], indicating a dynamic alteration of MSI phenotype during cancer development [21]. Further study is required to understand the MSI-H and its association with different stages of cancer development. Moreover, nine samples from Lynch syndrome patients were included to evaluate the effectiveness of MSI detection in Lynch syndrome who suffered from CRC in our cohort. It was not implied the frequency of Lynch syndrome in CRC population with and/or without MSI-H feature because these samples were not randomly selected.

**Table 1:** Sample demographics.

Characteristic	Number of patient	Percent (%)
Gender		
Female Male	39 58	40.0 60.0
Age		
< 50 ≥ 50, < 60	19 18	19.6 18.6
≥ 60, < 70	32	33.0
≥ 70	28	28.9
Tumor stage (T)		
1	6	6.2
2	12	12.4
3	66	68.0
4	13	13.4
Lymph node stage (N)		
0	61	62.9
1	25	25.8
2	11	11.3
Distant metastases (M)		
0	94	96.9
1	3	3.1
Disease stage		
I	13	13.4
II	44	45.4
III	37	38.1
IV	3	3.1
Tumor size		
< 4 cm	44	45.4
5–9 cm	49	81.4
>10 cm	4	4.1
Differentiation grade		
Poorly differentiated	4	4.1
Moderately differentiated	68	70.1
High differentiated	24	24.7
Unknown	1	1.0
Vascular invasion		
No	96	99.0
Yes	0	0
Unknown	1	1.0

**Table 2:**
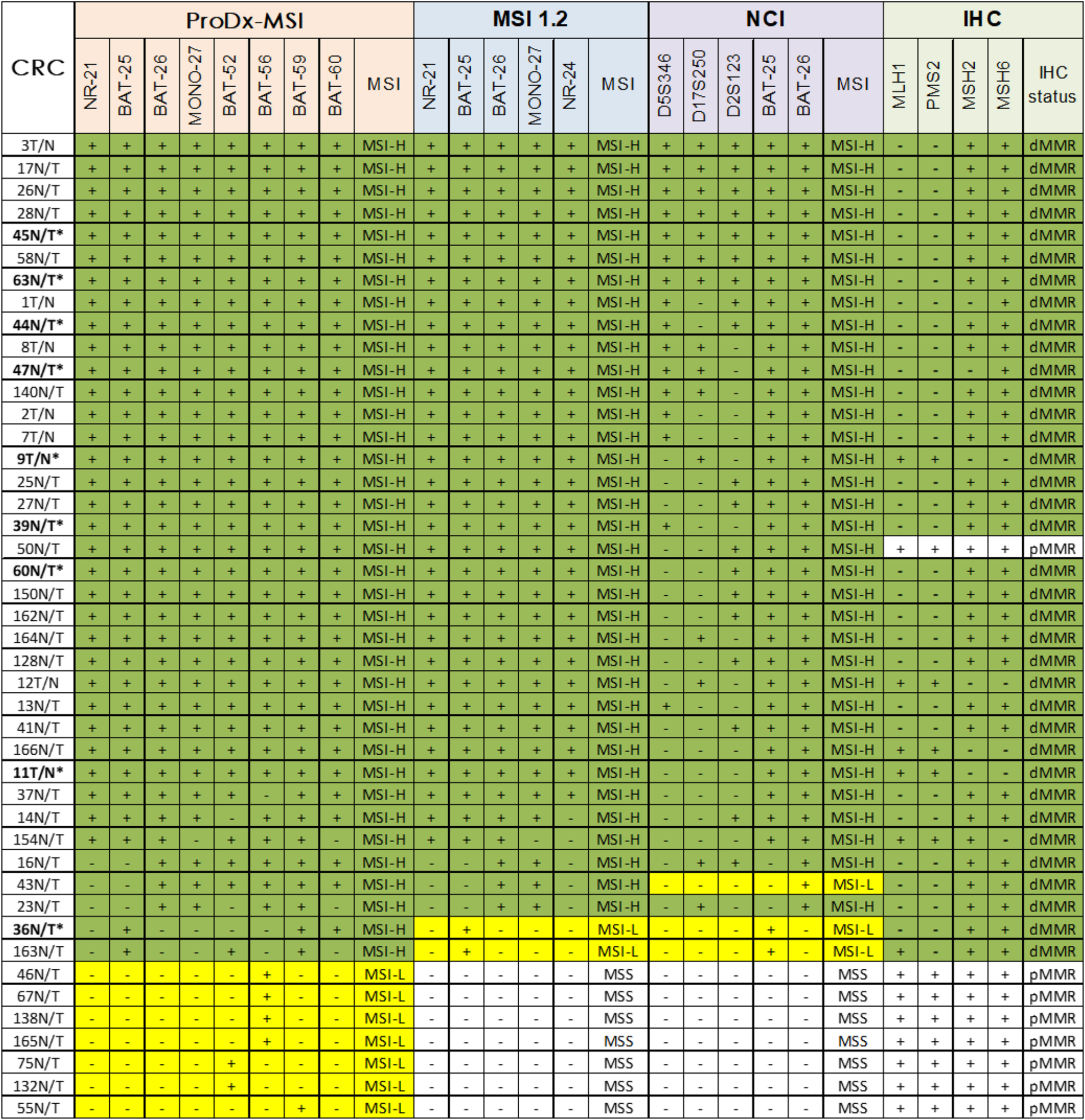
Comparison of MSI results for colorectal cancers with 3 assay panels

The MSI status classifications of tumor samples scored by the ProDx® MSI, the MSI 1.2, or the NCI panel. Samples were classified as MSI-H(green) when two or more markers were unstable, as MSI-L (yellow) when only one marker was unstable, and MSS when there was no any unstable marker. IHC staining scores were also listed in the right. The MSS samples by all panels and with intact MMR staining were not shown. “+” indicates MSI marker stable or MMR-IHC staining proficient (pMMR) ; “−” indicates MSI marker unstable or IHC staining deficient (dMMR). MMR-IHC deficient samples are also in green. Samples marked with “*”were Lynch syndrome cases that carried germline pathogenic MMR gene mutations

**Table 3:** MSI phenotypes grouped by the cancer development stage

Disease stage	MSI-H	MSI-L	MSS	Total
I	7 (53.8%)	2 (15.4%)	4 (30.8%)	13
II	19 (43.2%)	4 (9.1%)	21 (47.7%)	44
III	11 (29.7%)	1 (2.7%)	25 (67.6%)	37
IV	0	0	3 (100%)	3
Sum	37	7	53	97

**Table 4:** The sensitivity and specificity for each MSI microsatellite marker compared with MMR-IHC in 97 colorectal cases

	True poss	False poss	False neg	True neg	Sensitivity (%)	Specificity (%)
ProDx^®^ MSI						
BAT-60	33	1	3	60	91.7	98.4
BAT-59	36	2	0	59	100	96.7
BAT-56	33	5	3	56	91.7	91.8
BAT-52	33	3	3	58	91.7	95.1
NR-21	31	1	5	60	86.1	98.4
BAT-25	33	1	3	60	91.7	98.4
BAT-26	34	1	2	60	94.4	98.4
MONO-27	33	1	3	60	91.7	98.4
MSI 1.2						
NR-21	31	1	5	60	86.1	98.4
NR-24	29	1	7	60	80.6	98.4
BAT-25	33	1	3	60	91.7	98.4
BAT-26	34	1	2	60	94.4	98.4
MONO-27	33	1	3	60	91.7	98.4
NCI panel						
D5S346	16	0	20	61	44.4	100
D17S250	15	0	21	61	41.7	100
D2S123	18	1	18	60	50.0	98.4
BAT-25	33	1	3	60	91.7	98.4
BAT-26	34	1	2	60	94.4	98.4
